# Comparative Biomechanical Evaluation of SDS and Nobel Ceramic Dental Implant Systems in Type II Artificial Bone: An In Vitro Study

**DOI:** 10.3390/bioengineering13101186

**Published:** 2026-10-12

**Authors:** Carlos Fernando Mourão, Mona Patel, Luiz Eduardo Juliasse, Tea Romasco, Bruno César de Vasconcelos Gurgel, Etyene Schnurr

**Affiliations:** 1 Department of Basic and Clinical Translational Sciences, Tufts University School of Dental Medicine, Boston, MA 02111, USA; 2 Department of Dentistry, Federal University of Rio Grande do Norte, Natal 59064-630, Brazil; 3 Department of Medical, Oral and Biotechnological Sciences, “G. d’Annunzio” University of Chieti-Pescara, 66100 Chieti, Italy; 4 Department of Clinical Studies, Swiss Biohealth Clinic, 8280 Kreuzlingen, Switzerland; 5 Department of Basic Science, Institute of Health, Federal Fluminense University, Nova Friburgo 28625-650, Brazil

**Keywords:** dental implants, ceramics, zirconium, dental materials, osseointegration, torsion, mechanical, temperature, bone substitutes, biomechanical phenomena

## Abstract

Ceramic dental implants have been proposed as metal-free alternatives to titanium implants. This in vitro study compared the biomechanical measurements and drilling-related surface temperatures of two commercial ceramic implant systems with different dimensions and drilling protocols: SDS 2.2 (3.8 × 11 mm) and Nobel 2.2 (4.2 × 12 mm). Twenty-six implants per system were placed in type II polyurethane artificial bone. Outcomes included insertion torque, immediate removal torque, resonance frequency analysis in two perpendicular directions, and initial and final drill-surface temperatures. Observed insertion torque was 43.04 ± 5.38 Ncm for SDS and 31.27 ± 6.07 Ncm for Nobel; removal torque was 46.54 ± 5.47 and 28.31 ± 10.07 Ncm, respectively. SDS also had higher observed ISQ values in both directions. Mean final drill-surface temperatures were approximately 52 °C for SDS Drill 2 and 64 °C for Nobel Drill 4. These readings do not establish intraosseous temperature or biological thermal injury. Because implant dimensions, macrogeometry, and drilling protocols differed and independent block replication was limited, the results describe complete commercial systems under the tested conditions. Specimen-level statistical comparisons are exploratory and do not establish a confirmatory system effect. Further testing with both systems randomized across multiple independent blocks and direct, time-resolved substrate temperature measurements is needed.

## 1. Introduction

Dental implants are widely recognized as a predictable treatment option for replacing missing teeth. Titanium and titanium alloys remain the most extensively documented implant materials because of their mechanical reliability, biocompatibility, and long-term clinical performance. Nevertheless, titanium may present esthetic limitations due to its gray color, particularly when the peri-implant mucosa is thin or when gingival recession occurs in the anterior region. This concern has contributed to the development and increasing clinical adoption of ceramic implant systems, especially zirconia-based implants [1,2].

Zirconia is currently used in implant dentistry mainly as yttria-stabilized zirconia. The addition of yttria stabilizes the tetragonal crystal phase, which is associated with transformation toughening and improved fracture toughness and flexural strength. Zirconia has also been described as exhibiting osseointegration and soft-tissue response properties comparable to titanium, while offering potential advantages in esthetics, reduced bacterial adhesion, and lower cytotoxicity compared with titanium implants [3]. Currently available zirconia implants are predominantly fabricated from 3 mol% yttria-stabilized tetragonal zirconia polycrystal (Y-TZP) or alumina-toughened zirconia (ATZ), and may be produced as machined, sandblasted, sandblasted and acid-etched, laser-modified, or sinter-and-slurry surfaces [2]. Some implants are additionally heat-treated to retain the tetragonal phase and decelerate low-temperature degradation [2].

The clinical evidence for zirconia implants has expanded in recent years. In a systematic review and meta-analysis including 25 clinical studies, 4017 zirconia implants, and 2083 patients, Mohseni et al. [1] reported a 10-year cumulative survival rate of 95.1%. The same review reported that the mean marginal bone loss ranged from 0.632 to 2.060 mm during observation periods extending up to 132 months. However, the authors emphasized that zirconia implants remain less extensively studied than titanium implants, and that fractures have been reported, particularly in narrow-diameter implants and discontinued implant systems [1]. Recent prospective clinical evidence with two-piece ceramic implant systems has reported encouraging short-term performance: in a 12-month prospective trial of a two-piece Y-TZP implant restored both immediately and conventionally, da Silva et al. [4] reported a survival rate of 98.5% and mean marginal bone loss of approximately 0.24–0.27 mm at the mesial and distal sites. By contrast, Trimpou et al. [5], in a prospective case series of immediately restored, surface-modified two-piece zirconia implants, reported a 12-month survival of 80%, suggesting that immediacy protocols with ceramic implants may be associated with an increased risk of early implant losses and underscoring the importance of adequate primary mechanical stability at placement.

Implant design appears particularly relevant for zirconia implants. A systematic review evaluating the relationship between zirconia implant design and clinical outcomes reported promising five-year outcomes for one-piece zirconia implants, with success and survival rates ranging from 95% to 98.4% across the included prospective studies. The same review suggested that acid-etched roughened zirconia surfaces may be associated with more favorable outcomes than other surface designs. However, the authors also noted that the available evidence is limited and that high-quality comparative studies evaluating one-piece and two-piece zirconia implants with different diameters, lengths, and surface characteristics are still needed [3]. An integrative review correlating clinical, preclinical, and in vitro data on zirconia implant surfaces concluded that the lowest mean bone loss in clinical studies was reported for sandblasted and subsequently etched surfaces, followed by sinter-and-slurry treatments and sandblasted surfaces, but found no consistent correlation between surface structure and outcomes across the three study types, underlining the need for standardized procedures [2].

Primary stability is a key determinant of early implant performance. It is defined as the immediate mechanical engagement between the implant and the surrounding bone at the time of placement. This initial mechanical stability is influenced by implant design, implant diameter and length, surface characteristics, bone density, bone quantity, and the surgical protocol used for osteotomy preparation. During healing, primary mechanical stability gradually decreases as bone remodeling occurs, whereas secondary biological stability increases as osseointegration progresses [6].

Insertion torque and resonance frequency analysis (RFA) are among the most commonly used methods for assessing primary stability. Insertion torque reflects the rotational resistance encountered during implant placement, whereas RFA reflects the stiffness of the implant–bone or implant–substrate complex and is expressed as ISQ values. Although both methods are clinically and experimentally useful, they do not measure the same biomechanical phenomenon and should not be interpreted as interchangeable [6,7,8].

This distinction is supported by recent in vitro evidence. Rosas-Díaz et al. [8] evaluated 120 implants placed in polyurethane blocks equivalent to type II-A bone using different under-milling protocols. The authors reported that a 0.8 mm under-milling protocol produced significantly higher insertion torque values than a 0.2 mm protocol, whereas ISQ values did not differ significantly among the tested under-milling protocols. Their multivariate analysis reported partial η^2^ values of 0.639 for the compression protocol, 0.270 for implant diameter, and 0.121 for implant length for the combined insertion torque and ISQ outcomes [8].

Similarly, Brunel et al. [7] evaluated 600 implants placed in low-density polyurethane blocks and reported a weak inverse correlation between insertion torque and ISQ. In their global analysis, insertion torque explained only 8.2% of the variability in ISQ, reinforcing the interpretation that insertion torque and ISQ provide complementary but partially independent information about implant primary stability [7]. In a comparative in vitro study using bovine ribs, Arlucea et al. [9] reported that zirconia implants achieved significantly higher insertion torque (29.05 ± 11.46 Ncm) and ISQ (69.43 ± 6.63) than dimensionally identical titanium implants (23.00 ± 7.03 Ncm and 62.10 ± 10.61, respectively), but micromovement under load was comparable between the two materials. The authors attributed the higher RFA values for zirconia partly to its intrinsic stiffness, with a Young’s modulus approximately twice that of titanium (≈200 vs. ≈110 GPa), and cautioned against extrapolating titanium-based primary-stability decision thresholds directly to zirconia implants [9].

Thermal changes during osteotomy preparation are another important variable in implant site preparation. Mechanical drilling can generate heat in the bone adjacent to the osteotomy, and excessive temperature elevation may compromise bone viability. The commonly cited threshold for thermal bone injury is approximately 47 °C maintained for one minute. Although modern drilling protocols often remain below this value when performed adequately, heat generation is influenced by multiple factors, including bone density, drill design, drill diameter, drilling depth, rotational speed, feed force, drilling pattern, irrigation, drill wear, and the use of surgical guides [6,10,11].

A systematic review on guided implant drilling reported that surgical guides may increase heat generation during osteotomy preparation, potentially because guide sleeves can limit direct irrigation access to the drilling site. Nevertheless, the temperature rise in the included studies generally remained below the necrosis threshold, and heat generation was also influenced by irrigation temperature, drill length, drill diameter, and drilling speed [11]. A scoping review of heat generation during implant placement further emphasized that thermal behavior is multifactorial and identified irrigation and bone hardness as major determinants during the osteotomy phase [10]. In experimental studies, Abuhajar et al. [12] reported greater temperature increases with limiting than with non-limiting surgical guides. Gehrke et al. [13] found that a single-bur protocol did not generate more heat than conventional sequential drilling under the tested conditions. Baris et al. [14] found that cooling method and hand pressure affected temperature changes during osteotomy preparation.

Polyurethane bone blocks are commonly used in preclinical implant research because they provide standardized density and reduce biological variability. They have been recognized as useful materials for comparative testing of orthopedic devices and bone screws, and they allow controlled evaluation of implant placement mechanics across different substrate densities [15]. However, artificial bone models reproduce only selected mechanical aspects of human bone and do not simulate vascularity, cellular healing, remodeling, inflammation, or osseointegration. Findings from artificial bone studies should therefore be interpreted as preclinical biomechanical evidence rather than as direct predictors of clinical performance.

Although the biomechanical behavior of titanium implants in artificial-bone models has been extensively documented, comparative in vitro data on contemporary commercial ceramic implant systems remain comparatively scarce. The few available comparative studies tend to evaluate ceramic against titanium implants rather than directly comparing two commercial ceramic systems with different macrogeometries, dimensions, and manufacturer-specific drilling sequences. This represents a relevant knowledge gap, given that primary mechanical stability and thermal behavior in ceramic systems are jointly influenced by implant design, drilling protocol, and material stiffness [2,5,9].

The present in vitro study was designed to compare the biomechanical behavior of two commercial ceramic implant systems, SDS 2.2 and Nobel 2.2, inserted into standardized type II artificial bone. The study was conceived to provide controlled preliminary biomechanical evidence for these implant systems while acknowledging that biological osseointegration and clinical survival cannot be inferred from this model.

## 2. Materials and Methods

### 2.1. Study Design

This was an in vitro comparative biomechanical study evaluating two commercially available ceramic dental implant systems inserted into standardized type II polyurethane artificial bone. The evaluated outcomes were drill-surface temperature variation during osteotomy preparation, insertion torque, reverse torque resistance, and resonance frequency analysis in two perpendicular directions. The experimental protocol was developed at Tufts University School of Dental Medicine.

Because this study was performed exclusively in artificial bone blocks and did not involve human participants, human tissues, animals, or identifiable biological material, institutional review board approval was not required.

### 2.2. Implant Systems

Two ceramic implant systems were tested. The SDS group consisted of SDS 2.2 implants measuring 3.8 × 11 mm (SDS Swiss Dental Solutions AG, Kreuzlingen, Switzerland). The Nobel group consisted of Nobel 2.2 implants measuring 4.2 × 12 mm (Nobel Biocare AG, Kloten, Switzerland). Each group included 26 implants, for a total sample of 52 implants (Table 1).

### 2.3. Artificial Bone Model

**Figure 1:**
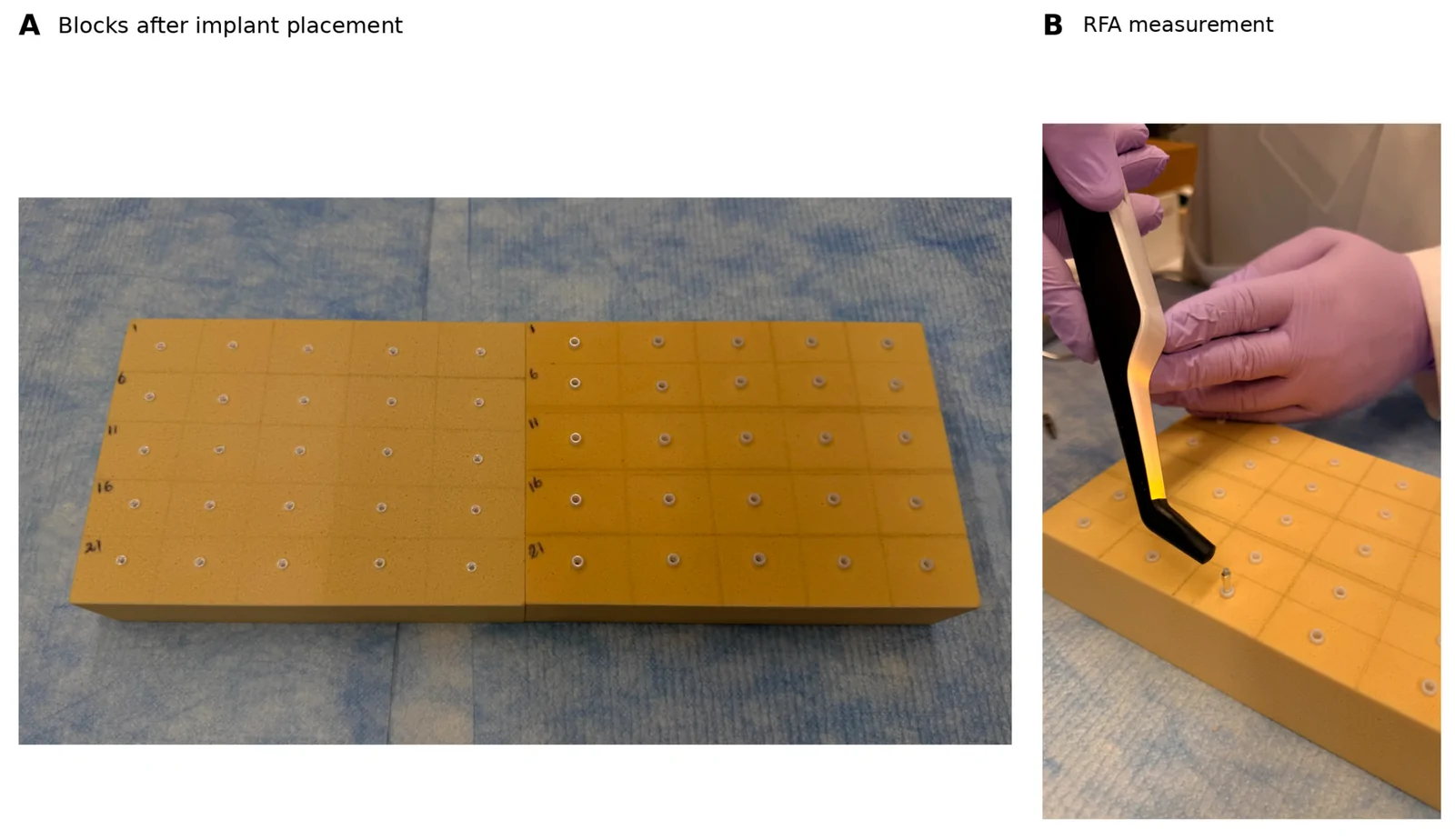
Photographs of the experimental specimens and resonance frequency analysis. (**A**) Two polyurethane blocks after placement of 25 implants each: Nobel on the left and SDS on the right. One additional implant from each system was placed in a shared third block. (**B**) Osstell RFA measurement on an SDS implant, with the SmartPeg seated in the implant. Panel (**A**) documents specimen arrangement, and panel (**B**) documents the measurement configuration; these photographs do not show the complete support or fixture used during torque testing.

All implants were inserted into standardized type II polyurethane artificial bone blocks (Sawbones, Vashon Island, WA, USA; 40 × 130 × 180 mm; 30 PCF (0.48 g/cm^3^)). Twenty-five SDS implants were placed in one block and 25 Nobel implants in a second block; one additional implant from each system was placed in a shared third block. The first two blocks after placement and an example of RFA measurement are shown in Figure 1. The implant was the unit of measurement, whereas the block was the substrate replication unit.

The use of artificial bone reduced biological variability, but it did not eliminate possible dependence among sites in the same block. This model is consistent with previous in vitro implant studies using polyurethane substrates to evaluate insertion torque, ISQ, and removal torque [15,16].

### 2.4. Osteotomy Preparation and Thermal Assessment

Osteotomies were prepared in type II polyurethane artificial bone using the system-specific preparation sequences selected in accordance with each manufacturer’s instructions for implant placement. All implant-related procedures were performed by the same operator. The SDS protocol comprised four sequential drilling steps, and the Nobel protocol comprised five. The recorded rotational speeds were 1000 rpm for SDS Steps 1 and 2 and 300 rpm for Steps 3 and 4. For Nobel, the recorded speeds were 800 rpm for Steps 1 and 2, 600 rpm for Steps 3 and 4, and 15 rpm for Step 5. Drilling depth, drilling duration, and the thermal camera frame rate were not recorded, which limits the reproducibility of the thermal measurements.

Initial and final drill-surface temperatures were recorded using a thermal imaging camera (TOPDON USA Inc., Rockaway, NJ, USA; TC005; spectral range, 8–14 µm). Temperature was read at the maximum-temperature location indicated by the red cursor on the exposed drill surface. The supplied thermograms display an emissivity setting of 0.98. This approach measured the drill surface and did not directly measure the temperature within the polyurethane block. The conventionally cited bone-injury temperature of approximately 47 °C maintained for one minute is therefore considered only as biological context [6,11].

For each drilling step, initial and final drill-surface temperatures were recorded, and the change (ΔT) was calculated as final minus initial. These observations do not establish the maximum temperature attained during the complete drilling event, the time to maximum temperature, or the duration of exposure at a specified temperature.

Heat generation during implant osteotomy is influenced by bone density, drill design and diameter, drilling depth and force, drilling pattern, irrigation, and drill wear. These variables are important for interpreting the thermal measurements [10,11].

### 2.5. Implant Placement and Insertion Torque

After osteotomy preparation, implants were inserted into the artificial bone blocks using the corresponding implant motor and placement protocol. The implant placement procedures were carried out using a commercial implant motor system (ProImplant, Bien-Air Dental SA, Bienne, Switzerland) equipped with an MX-i LED brushless micromotor (Bien-Air Dental SA, Bienne, Switzerland) and a 20:1 reduction contra-angle handpiece (CA 20:1 L, Bien-Air Dental SA). The motor torque limit during implant insertion was set to 70 Ncm. Insertion torque (IT) was recorded in newton-centimeters (Ncm) with a digital torque meter (DTW series, Nextech Global Co., Ltd., Bangkok, Thailand), operated in peak-hold mode. The instrument was new and was acquired for this study. The meter was zeroed unloaded before each measurement. Readings were taken in N·m and converted to Ncm (1 N·m = 100 Ncm). The maximum peak IT required to seat each implant in its final position was used for analysis. Insertion torque was interpreted as a measure of rotational resistance during implant placement. Because IT is influenced by implant macrogeometry, implant dimensions, drilling protocol, substrate density, and implant-bed compression, it was analyzed together with ISQ rather than as a standalone measure of primary stability [7,8].

### 2.6. Resonance Frequency Analysis

Immediate implant stability was assessed using resonance frequency analysis (RFA) with an Osstell^®^ device (Osstell^®^ AB, Göteborg, Sweden) and manufacturer-compatible SmartPegs (Type 83—SDS and Type 90—Nobel). ISQ values were recorded immediately after implant placement. Measurements were obtained in two perpendicular directions corresponding to buccolingual (RFA-BL) and mesiodistal (RFA-MD) equivalents within the polyurethane block; the mean of three consecutive readings per direction was used for analysis. This approach is consistent with contemporary in vitro studies that use multiple directional readings to improve reproducibility and reduce measurement variability [7].

### 2.7. Reverse/Removal Torque

After initial stability assessment, reverse/removal torque (RT) was measured during implant retrieval using the same calibrated torque device employed for IT measurement. RT was defined as the peak torque required to initiate implant removal from the artificial bone block.

In this study, RT was interpreted exclusively as a mechanical retention parameter. Because the study was performed in artificial bone without a healing period, this outcome does not represent secondary stability, bone remodeling, bone-to-implant contact, or osseointegration. This terminology was adopted to preserve methodological accuracy and to avoid biological overinterpretation of an in vitro synthetic-substrate model. This conceptual distinction is consistent with reviews of zirconia implant osseointegration that emphasize that biological removal torque values reflect bone–implant interface strength after a healing period and depend strongly on the animal model employed [2].

### 2.8. Sample Size Calculation

The sample size was determined a priori using G*Power 3.1.9.4 (Heinrich Heine Universität Düsseldorf, Düsseldorf, Germany). The calculation used the MANOVA global effects formulation with the following parameters for the biomechanical parameters: 2 groups (SDS vs. Nobel), 4 response variables (IT, RT, RFA-BL, RFA-MD), α: 0.05, target power: 0.80, and Pillai’s trace multivariate test. The calculation yielded a minimum sample size of 42 implants (21 per group). Contrarily, for the drilling temperature, the sample size was calculated based on an independent-samples *t*-test, two-tailed, with an effect size of d = 0.80 derived from previously reported temperature differences during implant osteotomy [12,13,14]. With α = 0.05 and 80% power, a total sample size of 52 (26 per group) was required. This sample size is consistent with or exceeds those used in similar in vitro drilling temperature studies. Therefore, 26 implants per group were used for each measurement.

### 2.9. Statistical Analysis

All statistical analyses were performed with GraphPad Prism 9.0 (GraphPad Software, San Diego, CA, USA). Descriptive statistics were calculated from 26 records per system and are presented as mean ± standard deviation. Normality was assessed using the Shapiro–Wilk test. Insertion torque was compared between systems using a two-sided Welch’s *t*-test. Removal torque and resonance frequency measurements in the buccolingual and mesiodistal directions were compared using two-sided Mann–Whitney U tests, with asymptotic *p*-values and corrections for ties and continuity. Holm correction was applied across the four biomechanical comparisons. Effect sizes were reported as Hedges g for insertion torque and rank-biserial correlations for the Mann–Whitney comparisons. Between-group differences in means were accompanied by 95% confidence intervals calculated using the Welch formulation; these intervals estimate mean differences and are distinct from the rank-based effects. Variance heterogeneity was assessed using median-centered Levene tests.

For each drilling step, initial and final temperatures from the same drilling event were treated as paired observations. Final-minus-initial changes were analyzed using two-sided paired *t*-tests, with Holm correction across the nine thermal contrasts. Exact sign tests were performed as sensitivity analyses because some change distributions departed from normality. These analyses assessed within-step temperature changes and did not test differences between drilling steps or between systems.

Because independent block replication was limited, interpretation was primarily descriptive. All specimen-level tests and confidence intervals were considered exploratory and were not adjusted for block clustering. Confidence intervals were marginal and were not adjusted for multiplicity. Holm correction addressed multiple comparisons but did not account for dependence among sites within the same block. The reported analyses therefore do not establish a confirmatory or generalizable system effect.

## 3. Results

**Figure 2:**
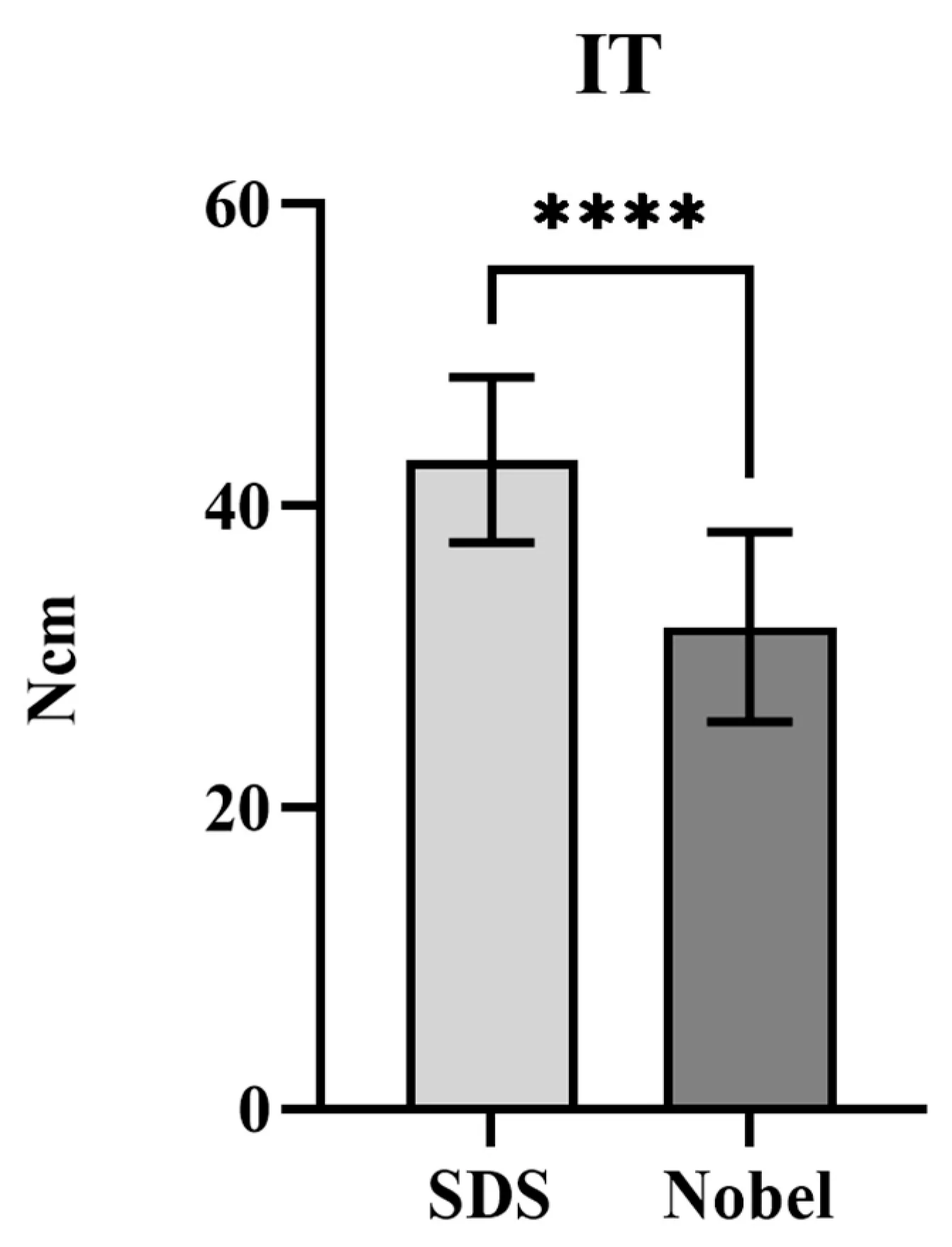
Original graphical summary of insertion torque (Ncm) for SDS and Nobel ceramic implants placed in type II polyurethane artificial bone. Bars represent group means and error bars represent SD. The unchanged graph and its embedded statistical annotation are retained from the earlier analysis; updated 26-record estimates and exploratory tests are reported in Section 3.1 and Table 2. The updated analyses do not adjust for block clustering. Four asterisks denote *p* < 0.0001 in the earlier graphical analysis.

**Figure 3:**
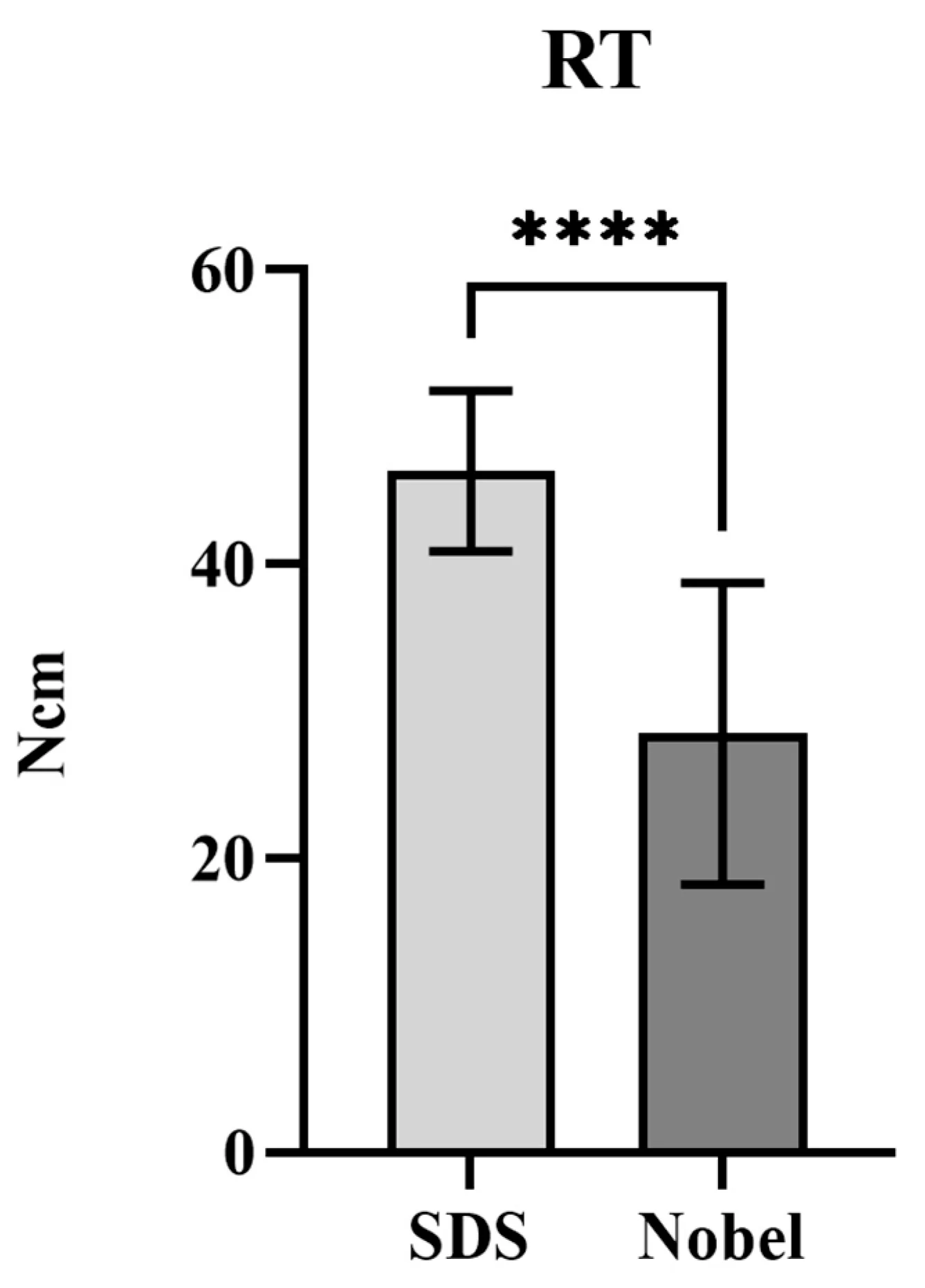
Original graphical summary of reverse/removal torque (Ncm) for SDS and Nobel ceramic implants placed in type II polyurethane artificial bone. Bars represent group means and error bars represent SD. The unchanged graph and its statistical annotation are retained from the earlier analysis; updated 26-record estimates and exploratory tests are reported in Section 3.2 and Table 2. These analyses do not adjust for block clustering. Four asterisks denote *p* < 0.0001 in the earlier graphical analysis.

**Figure 4:**
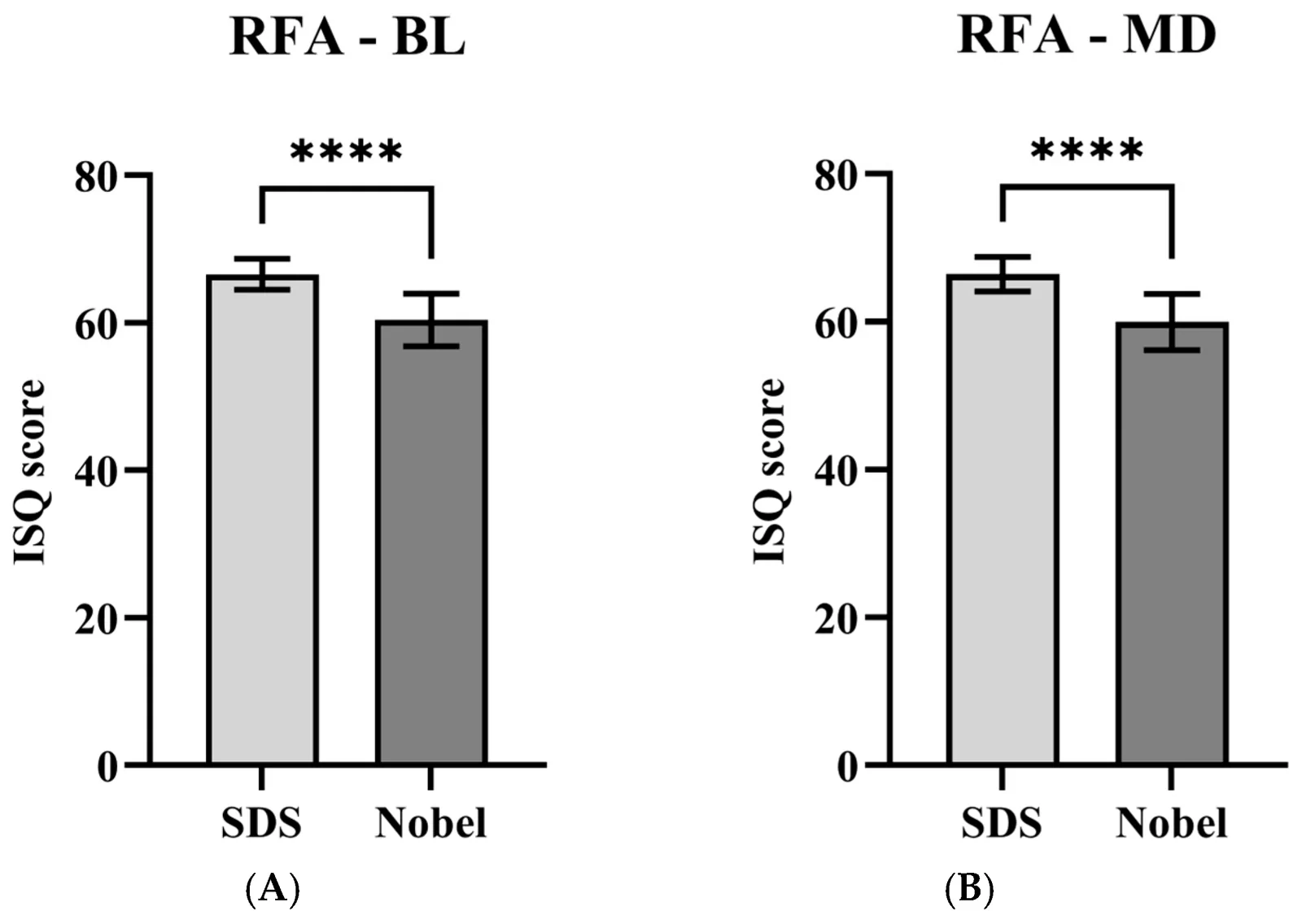
Original graphical summaries of resonance frequency analysis (ISQ) for SDS and Nobel ceramic implants: (**A**) buccolingual direction and (**B**) mesiodistal direction. Bars represent group means and error bars represent SD. The unchanged graphs and their statistical annotations are retained from the earlier analysis; updated 26-record estimates and exploratory tests are reported in Section 3.3 and Table 2. These analyses do not adjust for block clustering. Four asterisks denote *p* < 0.0001 in the earlier graphical analysis.

**Figure 5:**
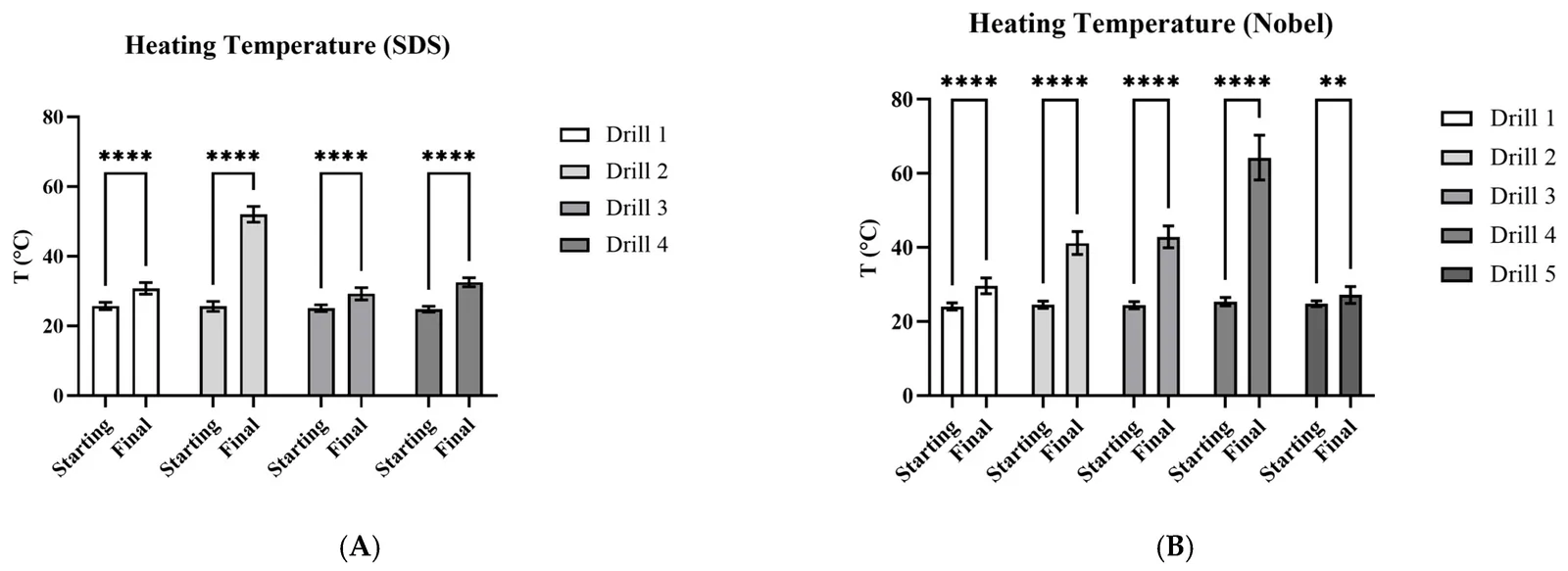
Original graphical summaries of initial and final drill-surface temperatures (°C) during sequential osteotomy preparation for (**A**) SDS (four steps) and (**B**) Nobel (five steps). Bars represent means and error bars represent SD. The unchanged graphs and embedded significance annotations are retained from the earlier analysis and have not been updated to the current 26-record paired analysis. Updated values and exploratory Holm-adjusted paired tests are given in Table 3; no mixed-effects or block-adjusted inference is claimed. Four asterisks denote *p* < 0.0001 and two asterisks denote *p* < 0.01 in the earlier graphical analysis.

The dataset contained 26 complete records per system for IT, RT, RFA-BL, RFA-MD, and all applicable initial/final drilling-temperature pairs. Numerical results were calculated directly from those records. The original study graphs are retained in Figure 2, Figure 3, Figure 4 and Figure 5; their earlier graphical summaries are not the source of the updated values in Table 2 and Table 3. Because block replication was limited, the results below are descriptive, and all reported specimen-level tests and confidence intervals are exploratory and unadjusted for block clustering.

### 3.1. Insertion Torque

Observed insertion torque was higher for SDS than for Nobel: 43.04 ± 5.38 versus 31.27 ± 6.07 Ncm, respectively. The mean difference (SDS minus Nobel) was 11.77 Ncm (exploratory 95% CI, 8.57 to 14.96); Hedges g = 2.02. Welch’s t = 7.400, df = 49.28; raw *p* = 1.54 × 10^−9^, Holm-adjusted *p* = 6.17 × 10^−9^. Shapiro–Wilk results were W = 0.9378, *p* = 0.1191 for SDS and W = 0.9637, *p* = 0.4691 for Nobel (Figure 2).

### 3.2. Removal/Reverse Torque

Observed removal torque was 46.54 ± 5.47 Ncm for SDS and 28.31 ± 10.07 Ncm for Nobel. The mean difference was 18.23 Ncm (exploratory mean-difference 95% CI, 13.68 to 22.78). Nobel removal-torque measurements were more dispersed than SDS measurements. The Mann–Whitney U statistic for SDS was 649.0; rank-biserial correlation = 0.920; raw *p* = 1.22 × 10^−8^, Holm-adjusted *p* = 1.84 × 10^−8^. Shapiro–Wilk results were W = 0.9550, *p* = 0.3030 for SDS and W = 0.8096, *p* = 0.0003 for Nobel. Mean RT exceeded mean IT for SDS but was lower than mean IT for Nobel; these descriptive mean relationships do not establish a within-implant torque difference (Figure 3).

### 3.3. Resonance Frequency Analysis

RFA values were obtained in two perpendicular directions: buccolingual (RFA-BL) and mesiodistal (RFA-MD). SDS had higher observed ISQ values in both directions:

- **RFA-BL:** 66.58 ± 2.06 ISQ for SDS versus 60.15 ± 3.73 ISQ for Nobel; mean difference, 6.42 ISQ (exploratory mean-difference 95% CI, 4.73 to 8.11). Mann–Whitney U for SDS = 654.5; rank-biserial correlation = 0.936; raw *p* = 6.12 × 10^−9^, Holm-adjusted *p* = 1.84 × 10^−8^. Shapiro–Wilk results: SDS W = 0.9472, *p* = 0.1996; Nobel W = 0.8365, *p* = 0.0008.
- **RFA-MD:** 66.46 ± 2.27 ISQ for SDS versus 59.77 ± 3.84 ISQ for Nobel; mean difference, 6.69 ISQ (exploratory mean-difference 95% CI, 4.93 to 8.46). Mann–Whitney U for SDS = 652.5; rank-biserial correlation = 0.930; raw *p* = 7.64 × 10^−9^, Holm-adjusted *p* = 1.84 × 10^−8^. Shapiro–Wilk results: SDS W = 0.8640, *p* = 0.0027; Nobel W = 0.8782, *p* = 0.0053.

ISQ group means were numerically similar between directions for each system (Figure 4). These descriptive similarities do not establish directional equivalence or symmetric implant–substrate stiffness.

### 3.4. Drilling Temperature

Mean initial drill-surface temperatures ranged from approximately 24 to 26 °C. Final drill-surface temperatures varied across drilling steps within each system (Figure 5). Exploratory paired analyses showed positive mean changes for all nine steps, but these tests do not account for block clustering.

For the SDS protocol (4 drilling steps), the initial and final drill-surface temperatures were: Drill 1: 25.65 ± 1.08 to 30.75 ± 1.61 °C; Drill 2: 25.56 ± 1.44 to 51.92 ± 2.31 °C; Drill 3: 25.03 ± 0.94 to 29.12 ± 1.76 °C; Drill 4: 24.79 ± 0.82 to 32.57 ± 1.29 °C.

**Figure 6:**
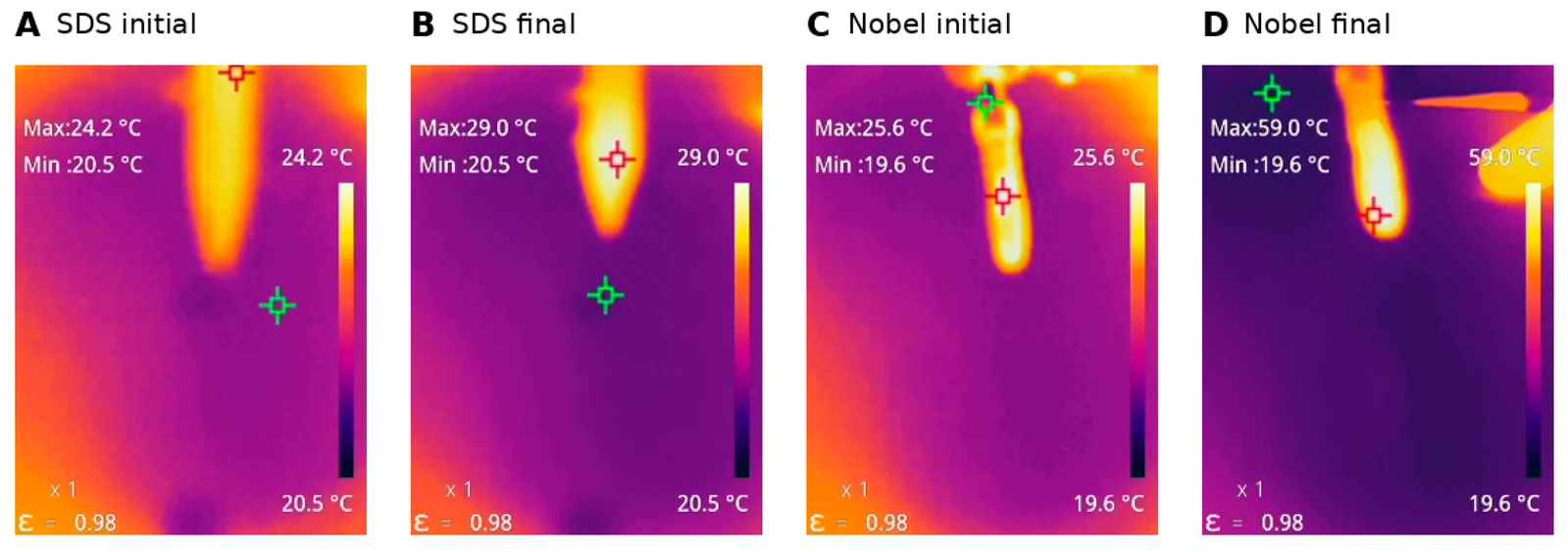
Representative infrared thermograms of the exposed fourth drill used in each osteotomy sequence: (**A**) SDS initial reading, 24.2 °C; (**B**) SDS final reading, 29.0 °C; (**C**) Nobel initial reading, 25.6 °C; and (**D**) Nobel final reading, 59.0 °C. The red cursor marks the maximum visible surface temperature in each image; the green cursor marks the minimum-temperature location. Original temperature displays, color scales, and emissivity indicators (ε = 0.98) are preserved. The examples illustrate temperature extraction and are not group means or time-resolved temperature records. They do not measure the temperature within the polyurethane block.

For the Nobel protocol (5 drilling steps), the initial and final drill-surface temperatures were: Drill 1: 24.07 ± 0.92 to 29.57 ± 2.13 °C; Drill 2: 24.57 ± 0.93 to 41.26 ± 3.07 °C; Drill 3: 24.40 ± 0.97 to 42.84 ± 2.91 °C; Drill 4: 25.38 ± 1.09 to 64.29 ± 5.89 °C; Drill 5: 24.81 ± 0.76 to 27.16 ± 2.23 °C. Detailed changes, confidence intervals, and exploratory Holm-adjusted *p*-values are reported in Table 3. All nine adjusted paired-test *p*-values were below 0.0001. The corresponding exact sign-test sensitivity analyses also remained below 0.0001 after Holm correction. Mean final temperatures exceeded 47 °C for SDS Drill 2 and Nobel Drill 4; two individual Nobel Drill 3 final readings also exceeded 47 °C despite its mean remaining below this value. These drill-surface measurements cannot demonstrate intraosseous thermal injury. Representative thermograms of the fourth drill in each sequence illustrate the maximum-temperature cursor used for the initial and final drill-surface readings (Figure 6).

## 4. Discussion

The present in vitro study compared two complete commercial ceramic implant systems, SDS 2.2 and Nobel 2.2, in type II polyurethane artificial bone. Observed IT, RT, and directional ISQ values were higher for SDS, and drill-surface temperature changes varied across the drilling sequences. Interpretation is descriptive because the implants were concentrated in two system-specific blocks, with only one implant from each system in the shared third block. The design therefore cannot separate a system effect from block effects with reliable independent replication.

### 4.1. Insertion Torque, Removal Torque, and Primary Stability

The approximately 12 Ncm difference in IT (≈43 vs. ≈31 Ncm) and the approximately 18 Ncm difference in RT (≈47 vs. ≈28 Ncm) between SDS and Nobel implants indicate substantially greater rotational engagement of the SDS system in the type II polyurethane substrate. The design does not isolate the contribution of material composition or individual system features. Possible contributors include macrogeometry, thread design, surface characteristics, drilling protocol (4 SDS drills vs. 5 Nobel drills), implant dimensions (3.8 × 11 mm vs. 4.2 × 12 mm), final osteotomy diameter, and implant–substrate interference, which could not be separated in the present design. In polyurethane substrates, the degree of bed compression generated by under-milling has been identified as a major determinant of IT. As shown by Rosas-Díaz et al. [8], the multivariate partial η^2^ values for IT and ISQ were 0.639 for the under-milling protocol, 0.270 for implant diameter, and 0.121 for implant length. However, because the final osteotomy dimensions, implant geometry, and degree of undersizing were not measured in the present study, it cannot be determined whether a more compressive drilling sequence, the implant macrogeometry, or other system-specific features accounted for the higher IT recorded for the SDS system; this explanation should therefore be regarded as a hypothesis to be tested in studies that control these variables independently.

Observed ISQ values were higher for SDS in both buccolingual and mesiodistal directions (≈66.5 vs. ≈60.0), and were similar in the two directions for each system. The parallel trends in IT and ISQ differences are informative because, as emphasized by Brunel et al. [7], insertion torque and ISQ are partially independent indices of primary stability: in their study of 600 implants, IT explained only 8.2% of the variability in ISQ. The fact that the SDS system showed higher values than the Nobel system in both indices simultaneously provides complementary descriptive evidence of greater observed mechanical engagement under the tested conditions. The ISQ pattern is also consistent with the comparative in vitro study by Arlucea et al. [9], in which zirconia implants achieved significantly higher ISQ than dimensionally identical titanium implants (69.43 vs. 62.10; *p* = 0.0296). Arlucea et al. [9] attributed part of this RFA difference to the intrinsic stiffness of zirconia (Young’s modulus ≈ 200 GPa, approximately twice that of titanium ≈ 110 GPa) and cautioned against extrapolating titanium-based primary-stability decision thresholds directly to zirconia implants. Because implant dimensions, macrogeometry, thread engagement, surface characteristics, material properties, and drilling protocols were not independently controlled, their individual contributions to the ISQ difference cannot be determined from this study. Block allocation also limits inference about a generalizable system difference.

Removal torque was higher than insertion torque in the SDS group (≈47 vs. ≈43 Ncm) but lower than insertion torque in the Nobel group (≈28 vs. ≈31 Ncm). RT was more dispersed in the Nobel group. These comparisons of group means do not establish a statistically significant paired IT–RT difference. In the absence of a healing period, RT measured in artificial bone reflects only the residual mechanical retention of the implant in the substrate after seating. As emphasized in the integrative review by Rohr et al. [2], biological removal torque values reflect bone–implant interface strength after healing and are strongly modulated by surface treatment and animal model; the in vitro RT values reported here should not be interpreted as proxies for osseointegration or secondary stability. Possible explanations for the different RT/IT relationships between the two systems include post-insertion settling of the polyurethane around the thread profile, micro-cracking of the substrate around the threads, and stress relaxation at the implant–substrate interface. However, none of these mechanisms was directly measured in the present study; they should therefore be regarded as hypotheses requiring dedicated testing (e.g., micro-computed tomography of the peri-implant substrate or time-dependent torque measurements), and the interpretation of RT should remain limited to mechanical retention in the artificial substrate.

### 4.2. Drilling Temperature

Mean drill-surface temperature increased at every step of both sequences. Mean final temperatures were approximately 29–33 °C for SDS Drills 1, 3, and 4 and approximately 52 °C for SDS Drill 2. For Nobel, the means were approximately 30 °C for Drill 1, 41 °C for Drill 2, 43 °C for Drill 3, 64 °C for Drill 4, and 27 °C for Drill 5. The conventionally cited bone-injury criterion is approximately 47 °C maintained for one minute [6,11]. However, the present readings were obtained from the drill surface, not within the substrate, and neither continuous temperature histories nor exposure duration were established. These measurements therefore cannot determine whether the surrounding substrate reached an injurious thermal exposure.

The measured mean final drill-surface temperature exceeded the conventionally cited temperature of 47 °C for SDS Drill 2 and Nobel Drill 4. This finding describes the drill surface and does not establish thermal injury in the substrate. Heat generation during osteotomy is multifactorial: Cimini et al. [10] and Saxena et al. [11] identify irrigation, substrate properties, drill geometry, speed, wear, and feed force as important variables. Differences in drill geometry, material removed per step, contact area, or irrigation access are possible explanations for the observed temperature changes, but their contributions were not measured independently in this study.

Several caveats apply to the thermal measurements. First, the thermal properties of the polyurethane substrate were not characterized, limiting extrapolation to clinical bone. Second, irrigation volume, flow rate, and irrigant temperature can substantially affect heat generation [10,11]. Third, infrared readings from an exposed drill cannot establish the temperature within the osteotomy. Fourth, the initial/final observations do not provide continuous temperature histories or a duration above 47 °C. Finally, paired measurements and multiplicity adjustment do not resolve the limited block replication. Future studies should document the operating conditions and measure substrate temperature directly over time.

### 4.3. Clinical and Prosthetic-Mechanical Context

The present biomechanical findings should be interpreted in the broader context of the available evidence on ceramic implants. Recent prospective clinical evidence on two-piece ceramic implant systems has reported variable short-term outcomes: da Silva et al. [4] observed a 12-month survival of 98.5% with mean marginal bone loss of approximately 0.24–0.27 mm in two-piece Y-TZP implants restored both immediately and conventionally, whereas Trimpou et al. [5] reported a 12-month survival of 80% in a prospective case series of immediately restored, surface-modified two-piece zirconia implants and explicitly cautioned that immediacy protocols may carry an increased risk of early implant losses if adequate primary mechanical stability is not achieved (an insertion torque ≥ 30 Ncm was used as the threshold for immediate restoration in that study). In the present in vitro study, both systems had mean IT values above 30 Ncm in the artificial substrate. These group means, obtained with limited block replication, do not establish clinical loading suitability or the probability that individual implants will meet a clinical torque criterion.

From a prosthetic-mechanical perspective, in vitro evidence on ceramic implant–abutment connections is also relevant. Fathi et al. [17] and da Silva Resende et al. [18] reported lower prosthetic screw-removal torque in the tested zirconia systems after cyclic loading than in the tested titanium systems. These findings support system-specific evaluation across the surgical and prosthetic phases rather than generalization across all ceramic interfaces.

The integrative review by Rohr et al. [2] further emphasized that surface topography descriptors such as Sa capture only the arithmetic mean roughness, and that current clinical, preclinical, and in vitro evidence on zirconia implant surfaces does not converge on a single optimal topography. Surface engineering remains an active research area: Aung et al. [19] showed that nano-rough Y-TZP implants functionalized with allylamine and fibronectin via glow-discharge plasma achieved higher bone-to-implant contact (62.35% vs. 53.79% at 12 weeks) and improved marginal bone gain in a rabbit femoral model, indicating that biological enhancement of the zirconia surface is a feasible route to improved osseointegration. The two systems compared in the present study were not benchmarked against surface-modified ceramic implants; further evaluation across modified zirconia surfaces would be informative.

### 4.4. Limitations

This study has some limitations. First, the implants differed in diameter, length, macrogeometry, surface characteristics, and drilling protocol; final osteotomy dimensions and the degree of undersizing were not measured. Individual system contributions cannot therefore be isolated. Second, 25 implants of each system shared a separate block and only one implant per system was placed in the shared third block. This allocation provides insufficient independent substrate replication for confirmatory between-system inference; specimen-level *p*-values and confidence intervals do not account for block dependence. Third, fatigue or cyclic loading was not evaluated, so durability under repeated mechanical loading was not established [17,18]. Fourth, surface topography was not independently characterized. Fifth, temperature was measured on the drill surface rather than within the substrate, and continuous thermal histories and exposure durations were not established. Sixth, polyurethane lacks vascularity, cellular healing, immune response, and remodeling; the measurements cannot predict osseointegration or clinical performance. Finally, only one reported type II substrate condition was tested. Future experiments should randomize both systems across multiple independent blocks, document site spacing and drilling conditions, and evaluate additional substrate densities.

## 5. Conclusions

Under the tested conditions, the SDS system had higher observed insertion torque, immediate removal torque, and directional ISQ values than the Nobel system. These are descriptive comparisons of complete commercial configurations with different implant dimensions and drilling protocols. Because most implants shared system-specific blocks and the shared third block contained only one implant per system, the results do not establish a confirmatory or generalizable system effect. Mean final drill-surface temperatures were approximately 52 °C for SDS Drill 2 and 64 °C for Nobel Drill 4; the means for the remaining steps were below 47 °C. These measurements do not establish intraosseous temperature or biological thermal injury. Future research should extend the evaluation of these ceramic implant systems to different bone densities and functional loading conditions, complemented by preclinical and clinical studies.

## Figures and Tables

**Table 1:** Ceramic implant systems evaluated in the study.

Group	Implant System	Material	Dimensions	Sample Size
SDS	SDS 2.2	Y-TZP zirconia	3.8 × 11 mm	26
Nobel	Nobel 2.2	ATZ	4.2 × 12 mm	26

The two commercial ceramic implant systems were inserted into standardized type II polyurethane artificial bone blocks and assessed for drill-surface temperature variation during osteotomy preparation, insertion torque (IT), reverse torque resistance (RT), and resonance frequency analysis in the buccolingual (RFA-BL) and mesiodistal (RFA-MD) directions, recorded as ISQ values. Note. Because the tested implants differ in both diameter and length, the study should be interpreted as a comparison between two commercial ceramic implant systems rather than as an isolated comparison of implant material composition, surface treatment, or macrogeometry alone. The total sample size was 26 implants per group (*n* = 52).

**Table 2:** Summary of biomechanical outcomes for SDS and Nobel ceramic implant systems in type II artificial bone (*n* = 26 per group).

Outcome	SDS (Mean ± SD)	Nobel (Mean ± SD)	Mean Difference and 95% CI	Exploratory Holm *p*
IT (Ncm)	43.04 ± 5.38	31.27 ± 6.07	11.77 (8.57–14.96)	6.17 × 10^−9^
RT (Ncm)	46.54 ± 5.47	28.31 ± 10.07	18.23 (13.68–22.78)	1.84 × 10^−8^
RFA-BL (ISQ)	66.58 ± 2.06	60.15 ± 3.73	6.42 (4.73–8.11)	1.84 × 10^−8^
RFA-MD (ISQ)	66.46 ± 2.27	59.77 ± 3.84	6.69 (4.93–8.46)	1.84 × 10^−8^

IT = insertion torque, RT = reverse/removal torque, RFA-BL = buccolingual resonance frequency analysis, RFA-MD = mesiodistal resonance frequency analysis, ISQ = implant stability quotient. Differences are SDS minus Nobel; confidence intervals estimate mean differences, including for outcomes analyzed with rank tests. All tests and intervals are exploratory and do not adjust for block clustering. Median-centered Levene *p*-values were 0.2345 (IT), 0.1941 (RT), 0.0892 (RFA-BL), and 0.0355 (RFA-MD). Holm correction addresses multiplicity and does not address the limited block replication.

**Table 3:** Initial and final drill-surface temperatures during osteotomy preparation (26 measured sites per system).

Group	Drill	Initial Temp. (°C)	Final Temp. (°C)	Mean ΔT and 95% CI	Exploratory Holm *p*
SDS	Drill 1	25.65 ± 1.08	30.75 ± 1.61	5.10 (4.34–5.87)	1.08 × 10^−12^
	Drill 2 *	25.56 ± 1.44	51.92 ± 2.31 *	26.36 (25.34–27.39)	2.95 × 10^−26^
	Drill 3	25.03 ± 0.94	29.12 ± 1.76	4.08 (3.34–4.82)	4.26 × 10^−11^
	Drill 4	24.79 ± 0.82	32.57 ± 1.29	7.78 (7.40–8.16)	7.63 × 10^−24^
Nobel	Drill 1	24.07 ± 0.92	29.57 ± 2.13	5.51 (4.79–6.22)	5.93 × 10^−14^
	Drill 2	24.57 ± 0.93	41.26 ± 3.07	16.70 (15.36–18.04)	8.84 × 10^−19^
	Drill 3	24.40 ± 0.97	42.84 ± 2.91	18.45 (17.30–19.60)	2.72 × 10^−21^
	Drill 4 *	25.38 ± 1.09	64.29 ± 5.89 *	38.91 (36.35–41.47)	8.63 × 10^−21^
	Drill 5	24.81 ± 0.76	27.16 ± 2.23	2.35 (1.41–3.29)	2.62 × 10^−5^

**Legend.** * Steps with mean final drill-surface temperature above 47 °C; these readings do not demonstrate substrate temperature or biological thermal injury. ΔT = final minus initial temperature. Values are mean ± SD, with exploratory marginal 95% confidence intervals for the paired mean changes. Holm correction covers nine paired contrasts; neither *p*-values nor intervals adjust for block clustering. Change distributions departed from normality for SDS Drill 3 (Shapiro–Wilk *p* = 0.0038), Nobel Drill 2 (*p* = 0.0098), and Nobel Drill 5 (*p* = 0.0057); the other six change distributions had *p* > 0.05. Exact sign-test sensitivity analyses supported the direction of the changes but do not resolve block dependence.

## Data Availability

The datasets used and/or analyzed during the current study are available from the corresponding author on reasonable request.
